# Toll-like receptor-2 deficiency enhances non-alcoholic steatohepatitis

**DOI:** 10.1186/1471-230X-10-52

**Published:** 2010-05-28

**Authors:** Chantal A Rivera, LaTausha Gaskin, Monique Allman, Jia Pang, Kristen Brady, Patrick Adegboyega, Kevin Pruitt

**Affiliations:** 1 Department of Molecular and Cellular Physiology, Louisiana State University Health Sciences Center, Shreveport, LA USA; 2 Department of Pathology, Louisiana State University Health Sciences Center, Shreveport, LA USA

## Abstract

**Background:**

Previously we reported that mice deficient in toll-like receptor 4 (TLR-4) signalling were protected from diet-induced non-alcoholic steatohepatitis (NASH). Another member of the toll-like receptor family, TLR-2, has been shown to play a role in lipid trafficking via uptake of diacylated lipoproteins. However, a role for TLR-2 in NASH has not been elucidated. The objectives of the current study were to examine the influence of dietary fat quality and TLR-2 on NASH pathogenesis.

**Methods:**

Steatohepatitis was induced in male Db, C57BL/6 and TLR-2^-/- ^mice by feeding an L-amino acid-defined diet that was deficient in methionine and choline (MCDD). Mice fed the base diet supplemented with methionine and choline (control diet; CD) were used as controls. To determine the role of fat quality, MCDD was enriched with polyunsaturated corn oil (PUFA) or coconut oil that is comprised mostly of saturated fat (SAFA); the total amount of each fat was 112.9 g/kg of diet. After 8 weeks of feeding CD or MCDD, hepatic steatosis, inflammation and necrosis were evaluated in histological sections. Total RNA was extracted from frozen liver samples and mRNA expression of TNFα, collagen α1, IL-10, peroxisome proliferator-activated receptor-γ (PPAR-γ), TLR-4, and CD14, was analyzed via real-time PCR. Protein levels of TLR-2 were analyzed by western blot.

**Results:**

Panlobular macrovessicular steatosis and diffuse leukocyte infiltration were noted in PUFA-fed Db mice. Histological scores demonstrated significantly less steatosis, inflammation and necrosis in SAFA-fed mice of all mouse strains. However, compared to wild type mice, hepatocellular damage was notably more severe in TLR-2^-/- ^mice. Consistent with histological findings, mRNA expression of TNFα was elevated by approximately 3-fold in TLR-2^-/- ^mice; PPAR-γ expression was blunted in this strain compared to wild type. Expression of the matrix protein collagen αI was also significantly higher in TLR-2^-/- ^mice, indicating a pro-fibrogenic state. Sensitivity to steatohepatitis due to dietary fat or TLR-2 deficiency correlated significantly with alterations in the expression of TLR-4 as well as the co-receptor CD-14.

**Conclusions:**

Our findings suggest that dietary saturated fat plays a protective role against MCDD-induced steatohepatitis, whereas TLR-2 deficiency exacerbated NASH. The mechanism underlying the response to dietary fat and TLR-2 likely involves altered signalling via the TLR-4 pathway.

## Background

For more than a decade the worldwide proportion of obese individuals has increased yearly. Along with rates of obesity, the incidence of co-morbid conditions such as type 2 diabetes, cardiovascular disease and liver disease have also increased. Non-alcoholic steatohepatitis (NASH) is a form of liver disease associated with obesity, and is characterized by the accumulation of lipid droplets in the cytoplasm of hepatocytes, mixed cell type inflammation, necrosis and often may involve some degree of fibrosis. The prevalence of NASH is reportedly as high as 40-100% of obese patients in some studies [1]. Initiating factors and underlying mechanisms of progression of this type of liver disease have not yet been elucidated.

The degree of adiposity in humans is attributed primarily to poor lifestyle habits such as excessive caloric intake. As a result, recent investigations have focused on elucidating the contribution of diet to the development of systemic pathologies. A positive correlation has been found to exist between the incidence of metabolic syndrome and the amount of total dietary fat intake of obese humans. One of the major constituents of the "western" diet (WD) typically consumed in the United States is saturated fat. A study using a simulation model based on hypothesized dietary changes reported that decreasing daily intake of saturated fat by 5 grams would reduce the number of obese people by 3.9 million, thereby markedly reducing costs of obesity-associated healthcare [2]. Indeed, the content of saturated fat correlates positively with the incidence of several co-morbid conditions such as type 2 diabetes and cardiovascular disease [3-7]. Studies performed in vitro have demonstrated the ability of saturated fatty acids to directly stimulate a pro-inflammatory phenotype [8-11], which likely contributes to disease pathogenesis. For example, addition of the saturated fatty acids palmitate and stearate to cultures of human pancreatic islets elicited the production of various pro-inflammatory cytokines and chemokines [12]. Palmitate similarly stimulated inflammation in endothelial cells [5]. Further studies in various cell types, including endothelial cells as well as macrophage cell lines, indicate that the observed inflammatory response is mediated by signalling via the toll-like receptor (TLR) pathway [10,13].

The TLR family of pattern recognition receptors is critical in host defence against invading pathogens. Of the approximately 13 mammalian TLRs, TLR-4 and TLR-2 have been studied extensively in the setting of obesity-associated inflammation and disease pathogenesis. These receptors respond to gram negative and gram positive organisms, respectively. Recently, we reported that mice deficient in TLR-4 signalling due to a spontaneous mutation were protected from diet-induced NASH [14]. Depletion of Kupffer cells via administration of liposome encapsulated clodronate significantly reduced TLR-4 expression and similarly blunted steatohepatitis. TLR-2 has been shown to play a role in lipid trafficking via uptake of diacylated lipoproteins [15], a process that requires CD36 [16]. Although a significant role for TLR-4 signalling in the progression of NASH has been established, a role of the closely-related TLR-2 pathway remains to be determined.

The focus of the present study was to investigate the potential interactions between dietary fat quality and TLR signalling. The major objectives were to 1) examine the influence of saturated fat relative to polyunsaturated fat; and 2) investigate the importance of TLR-2 in the pathogenic mechanisms underlying NASH. To this end, we compared the extent of steatohepatitis in wild type and TLR-2 deficient mice fed a methionine and choline deficient diet (MCDD) enriched with polyunsaturated corn oil or coconut oil as the saturated fat. In this model hepatic microvascular dysfunction and pronounced pathological changes develop within 3-4 weeks [17-19]. Based on previous findings of the pro-inflammatory roles of saturated fat and TLR-2 signalling that adversely effect the vasculature, we hypothesized that feeding saturated fat as the primary fat source would exacerbate NASH and that TLR-2 deficiency would protect against NASH pathogenesis.

## Methods

### Animal treatment

Male Db and C57BL/6 mice were purchased from Jackson laboratories at 4-6 weeks of age. The TLR-2^-/- ^mice were initially a gift from A. Akira (Tokyo, Japan) and were back-crossed on to the C57BL/6 background for 8 generations. Steatohepatitis was induced by feeding an L-amino acid-defined diet that was deficient in methionine and choline (MCDD). The base diet supplemented with methionine and choline was used as a control diet (CD). As outlined in Table 1, the MCDD diets were enriched with 112.9 grams of coconut oil (SAFA) or corn oil (PUFA). Mice were fed ad libitum for 8 weeks. The protocols used for handling mice were approved by the Louisiana State University Health Sciences Center Animal Care Committee and were in accordance with the guidelines set by the National Institutes of Health *Guide for Care and Use of Laboratory Animals*.

**Table 1:** Dietary Components.

Ingredient (g/Kg)	Control Diet	PUFA Diet	SAFA Diet
Cornstarch	419.1	384.7	384.7

Dyetrose	140	140	140

Sucrose	100	100	100

Cellulose	50	50	50

oil	70	112.9	112.9

Salt Mix #210030	35	35	35

Sodium Bicarbonate	6.4	6.4	6.4

Vitamin Mix#310025	10	10	10

Choline Bitartrate	2.5	0	0

### Assessment hepatic injury

After 8 weeks of feeding, a small section of each liver was preserved in zinc-buffered fixative and sections were stained with hematoxylin and eosin to assess hepatic injury. Steatosis, inflammation and necrosis were scored by one of the authors (P. A.) that was blinded to the study design. The absence of these histopathological features was scored as 0 and the most severe changes were given a score of 3.

### Reverse transcription and real-time PCR

Total RNA was extracted from frozen liver samples using the Qiagen RNeasy reagents. Each total RNA sample (500 ng) was reverse transcribed using TaqMan transcription buffer and multiscribe reverse transcriptase (Applied Biosystems; Foster City, CA). The relative mRNA expression of TNFα, IL-10, peroxisome proliferator-activated receptor-γ (PPAR-γ), collagen α1, TLR-4, and CD14 was analyzed using pre-developed assays for real-time PCR (Applied Biosystems). In a separate tube, ribosomal 18s was amplified as a reference. Gene expression was quantified using a comparative critical threshold (*C*_T_) method as described previously [20].

### Western blotting

Total protein (50 μg) was separated by gel electrophoresis and transferred to polyvinylidene fluoride membranes. Membranes were blotted with antibodies directed against TLR-2 (4°C overnight; Cell Signalling Technology; Danvers, MA) or β-actin (1 h at ambient temperature; BD Biosciences) then incubated with an isotype-specitific HRP-conjugated secondary antibody (1 h at ambient temperature). Target proteins were visualized using extended duration substrate detection reagents (Pierce) in a Chemidoc XRS documentation system (Bio-Rad Laboratories; Hercules, CA).

### Data Analysis

Statistical analysis was performed on histological scores using ANOVA on Ranks; the remaining data was analyzed using two-way ANOVA with p < 0.05 as the level of significance. For each parameter tested, n = 6 observations per group were analyzed.

## Results

### Effect of feeding unsaturated or saturated fat-enriched diet on steatohepatitis

**Figure 1:**
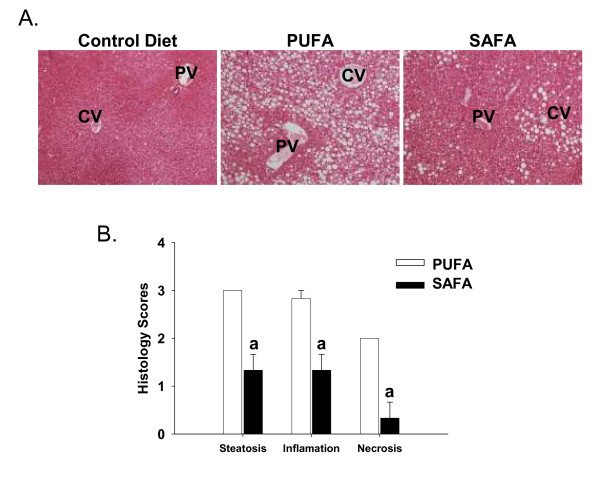
**Hepatic histopathology**. **(A)**. Representative H&E-stained photomicrographs of livers obtained from Db mice after 8 weeks of feeding control diet (CD), corn oil diet (PUFA) or coconut oil diet (SAFA). PV = portal vein; CV = central vein. **(B) **Summary of histology scores. The following scoring system was used for each parameter. 1 = mild; 2 = moderate; 3 = severe. Original magnification = 20×. Data was analyzed using Student's t test; ^a^p < 0.05 compared to PUFA.

The goal of the first set of experiments was to determine the extent of NASH pathogenesis in Db mice fed corn oil-enriched PUFA diet or SAFA that was enriched in coconut oil as the fat source. To induce robust features of NASH, both diets were deficient in methionine and choline. Panlobular macrovessicular steatosis and diffuse leukocyte infiltration was noted in PUFA-fed mice (Fig. 1). Histological findings were comparably less severe in the SAFA group. In fact, injury in this group was limited primarily to zone 3. A summary of the histological scores is presented in Figure 1B; the pathology observed was highly reproducible with minimal inter-animal variation. These scores demonstrate significantly less steatosis, inflammation and necrosis in SAFA-fed mice.

### Effect of diet on TNF-α and collagen α1 expression

**Figure 2:**
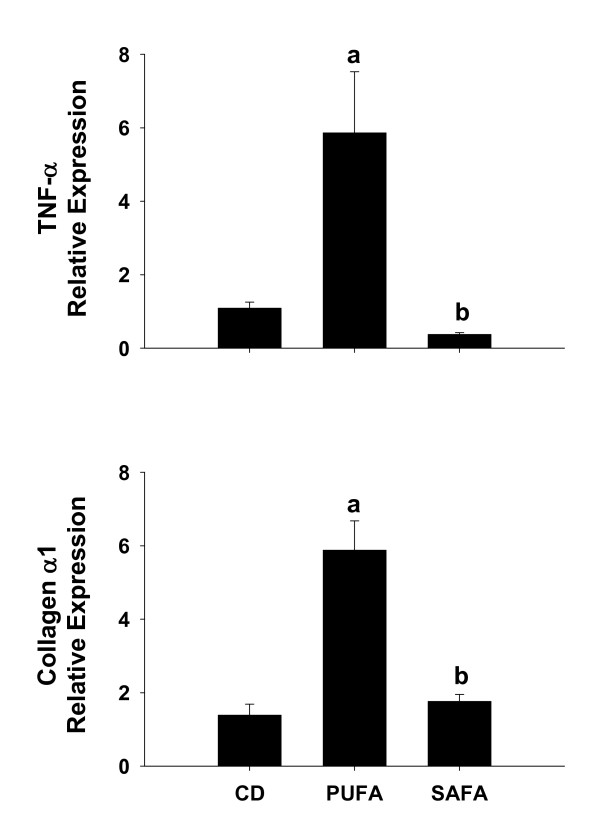
**Hepatic TNF-α and Collagen mRNA levels**. Expression of TNF-α and Collagen α1 mRNA was analyzed by real-time PCR and calculated relative to average values of mice fed control diet (CD) using a comparative C_T _method. Data are presented as mean ± SEM of 4 observations/group. Statistical comparisons were made using one-way ANOVA. ^a^p < 0.05 compared to mice fed CD; ^b^p < 0.05 compared to mice fed PUFA.

To index inflammation and fibrosis, mRNA expression of TNF-α and collagen α1 (Fig. 2) were measured using quantitative real-time PCR. Compared to Db mice fed CD, there was an approximately 6-fold increase in TNF-α and collagen in PUFA-fed mice. Increases in both of these parameters were prevented in mice that were fed SAFA.

### Effect of diet on toll-like receptor-4

**Figure 3:**
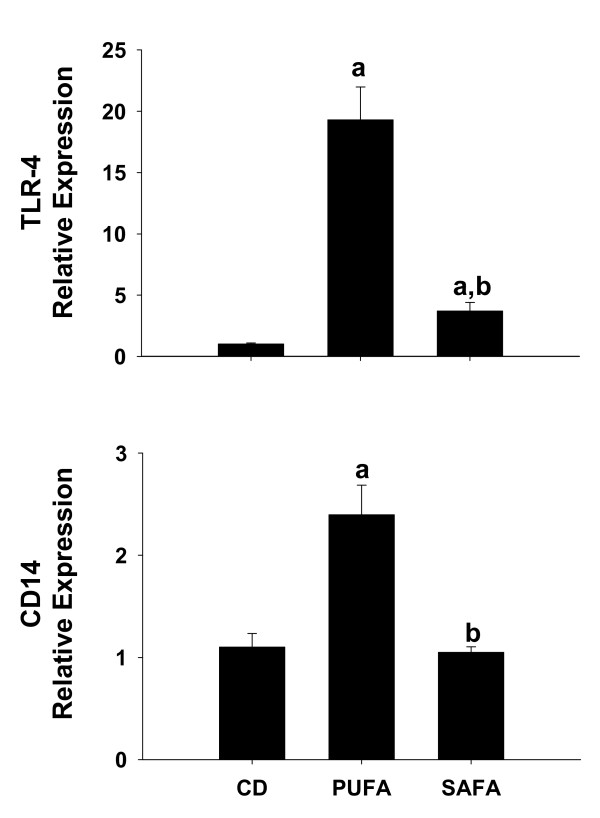
**Expression of TLR-4 and the co-receptor CD14**. For assessment of TLR-4 and CD14 expression, livers were collected from male Db mice fed control diet (CD), PUFA or SAFA for 8 weeks. Pre-developed assays for real-time PCR were used according to the manufacturer's instructions (Applied Biosystems). Expression of each target mRNA was calculated relative to average values in the CD group using a comparative C_T _method and presented as mean ± SEM of 4 observations/group. Statistical comparisons were made using one-way ANOVA. ^a^p < 0.05 compared to control diet (CD); ^b^p < 0.05 compared to mice fed PUFA.

We showed previously that TLR-4 signalling was critical for NASH pathogenesis [14]. In an attempt to determine if differences in TLR-4 expression might explain the blunted injury in mice fed SAFA, mRNA expression of TLR-4 and the co-receptor CD14 was examined. As expected, expression of TLR-4 and CD14 was enhanced significantly by approximately 2.5-fold and 20-fold, respectively, in mice fed the PUFA diet (Fig. 3). In contrast, feeding SAFA blunted increases in mRNA levels of both TLR-4 and CD14.

### Effect of diet on toll-like receptor-2

**Figure 4:**
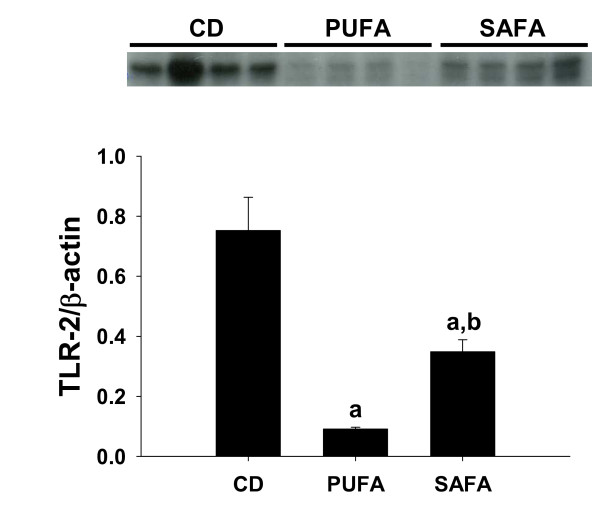
**Hepatic TLR-2 expression**. (**A**) Western blot analysis of hepatic TLR-2. n = 4 observations for each dietary group. **(B) **Densitometry of hepatic TLR-2 expression relative to β-actin. Data are mean ± SEM. ^a^p < 0.05 compared to control using one-way ANOVA.

Next, the influence of diet on the expression of TLR-2 was investigated. Western blot analysis revealed a dramatic reduction in protein levels of TLR-2 in PUFA-fed mice (Fig. 4). As summarized in Figure 4, the reduction in TLR-2 expression was markedly attenuated in mice fed the SAFA diet.

### Effect of diet on Steatohepatitis in TLR-2-/- mice

**Figure 5:**
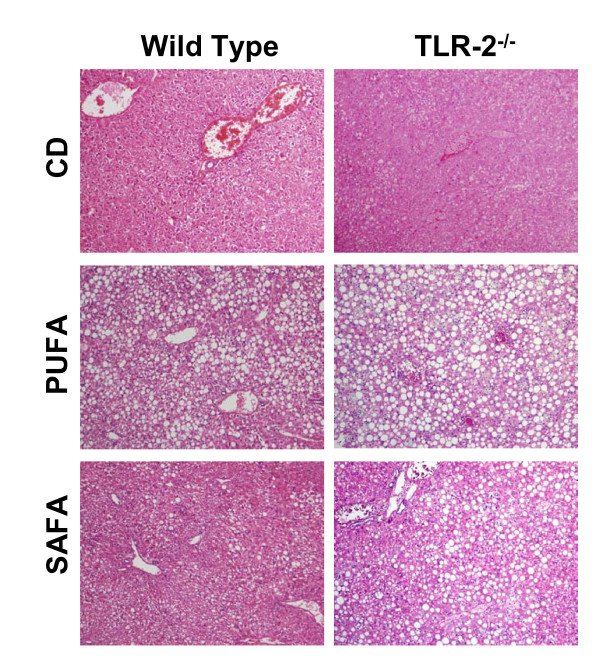
**Hepatic histopathology in TLR-2^-/- ^mice**. Representative H&E-stained photomicrographs of livers obtained from wild type and TLR-2^-/- ^mice after 8 weeks of feeding control diet (CD), PUFA or SAFA. PV = portal vein; CV = central vein. Original magnification = 32×.

Based on the opposing effects of the PUFA and SAFA diets on protein levels of TLR-2, subsequent experiments were designed to investigate what role TLR-2 might play in NASH pathogenesis. In this series of experiments C57BL/6 wild type and TLR-2^-/- ^mice were fed PUFA or SAFA for 8 weeks. Similar to findings in Db mice, C57BL/6 wild types fed PUFA displayed histological evidence of steatosis and injury (Fig. 5). These features were markedly reduced in SAFA-fed wild type mice (Fig. 5). Feeding either of these methionine/choline-deficient diets exaggerated NASH in TLR-2^-/- ^mice, resulting in panlobular steatohepatitis.

### Enhanced expression of inflammatory and fibrosis markers in toll-like receptor-2 deficient mice

**Figure 6:**
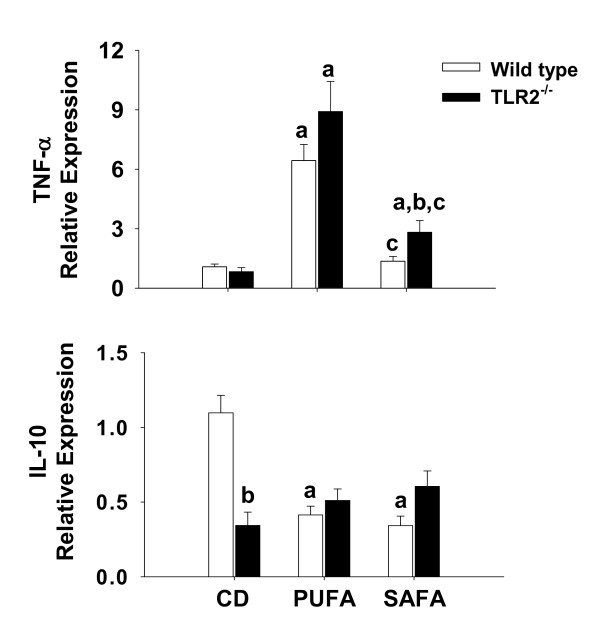
**Hepatic TNF-α and IL-10 mRNA levels in TLR-2^-/- ^mice**. Expression of TNF-α and IL-10 mRNA was analyzed by real-time PCR and calculated relative to average values in the control group using a comparative C_T _method. Data are presented as mean ± SEM of 5 observations/group. Statistical comparisons were made using two-way ANOVA and Tukey multiple comparisons test. ^a^p < 0.05 compared to strain-matched mice fed control diet (CD); ^b^p < 0.05 compared to diet-matched wild type (WT) mice; ^c^p < 0.05 compared to strain-matched mice fed PUFA.

Messenger RNA expression of the proinflammatory cytokine TNF-α as well as the anti-inflammatory cytokine IL-10 was assessed by real-time PCR to index the hepatic inflammatory state. Expression of TNF-α was similar among wild type and TLR-2^-/- ^mice fed PUFA (Fig. 6). In SAFA-fed mice, mRNA expression of this cytokine was higher by approximately 3-fold in TLR-2^-/- ^mice compared to diet-matched wild type mice. Feeding MCDD to wild type mice diminished IL-10 expression irrespective of the type of fat included in the diet (Fig. 6). In CD-fed mice, expression of this cytokine in TLR-2^-/- ^mice was approximately 3-fold lower than wild types; feeding MCDD had no further effect on IL-10 expression in the TLR-2 deficient mouse strain.

**Figure 7:**
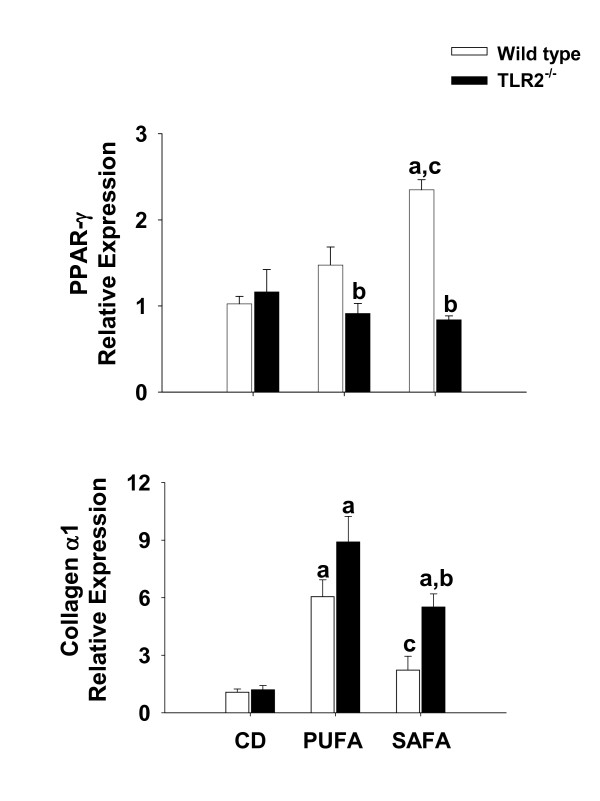
**Hepatic PPAR-γ and collagen α1 mRNA levels in TLR-2^-/- ^mice**. Expression of PPAR-γ and Collagen α1 mRNA was analyzed by real-time PCR and calculated relative to average values in the control group using a comparative C_T _method. Data are presented as mean ± SEM of 5 observations/group. Statistical comparisons were made using two-way ANOVA and Tukey multiple comparisons test. ^a^p < 0.05 compared to strain-matched mice fed control diet (CD); ^b^p < 0.05 compared to diet-matched wild type (WT) mice; ^c^p < 0.05 compared to strain-matched mice fed PUFA.

Similar to TNF-α, mRNA levels of the matrix protein collagen αI, a marker of hepatic fibrogenic potential, was highest in PUFA-fed mice of both strains; however TLR-2 deficiency significantly enhanced expression in mice fed SAFA when compared to diet-matched wild types (Fig. 7). To further index the response to diet and TLR-2 deficiency, mRNA levels of PPAR-γ were assessed. This receptor has been shown to have anti-inflammatory properties and to blunt fibrogeneis via regulation of stellate cell activation. Expression of this receptor was enhanced significantly in wild type mice fed SAFA (Fig. 7). Consistent with increased inflammation and fibrogenic potential observed in TLR-2^-/- ^mice, PPAR-γ expression in this mouse strain was significantly lower than diet-matched wild type mice fed PUFA or SAFA.

### Toll-like receptor-4 signalling is augmented in toll-like receptor-2 deficient mice

**Figure 8:**
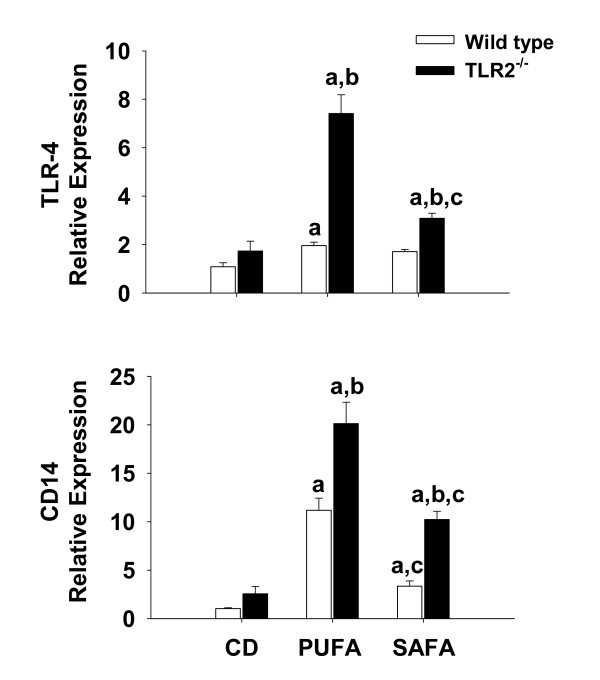
**Expression of TLR-4 associated signaling molecules**. For assessment of TLR-4 and CD14 expression, livers were collected from male C57BL/6 and TLR-2^-/- ^mice fed CD or MCDD for 8 weeks. Pre-developed assays for real-time PCR were used according to the manufacturer's instructions. Expression of each target mRNA was calculated relative to average values in the control group using a comparative C_T _method and presented as mean ± SEM of at least 4 observations/group. Statistical comparisons were made using two-way ANOVA and Tukey multiple comparisons test. ^a^p < 0.05 compared to strain-matched mice fed control diet (CD); ^b^p < 0.05 compared to diet-matched wild type (WT) mice; ^c^p < 0.05 compared to strain-matched mice fed PUFA.

To investigate activation of TLR-4 signalling, mRNA expression of components of the TLR-4 pathway were quantified via real-time PCR. Consistent with findings in the Db strain, SAFA attenuated the expression of TLR-4 and CD14. Compared to wild type mice, a significant increase in TLR-4 expression was observed in TLR-2^-/- ^mice fed PUFA or SAFA (Fig. 8). Expression of the TLR-4 co-receptor CD14 was also increased significantly by 2-fold and 4-fold, respectively, in TLR-2^-/- ^mice fed PUFA or SAFA when compared to diet-matched wild type mice (Fig. 8).

### Expression of inflammatory and fibrosis markers correlate with toll-like receptor-4 and CD14

**Figure 9:**
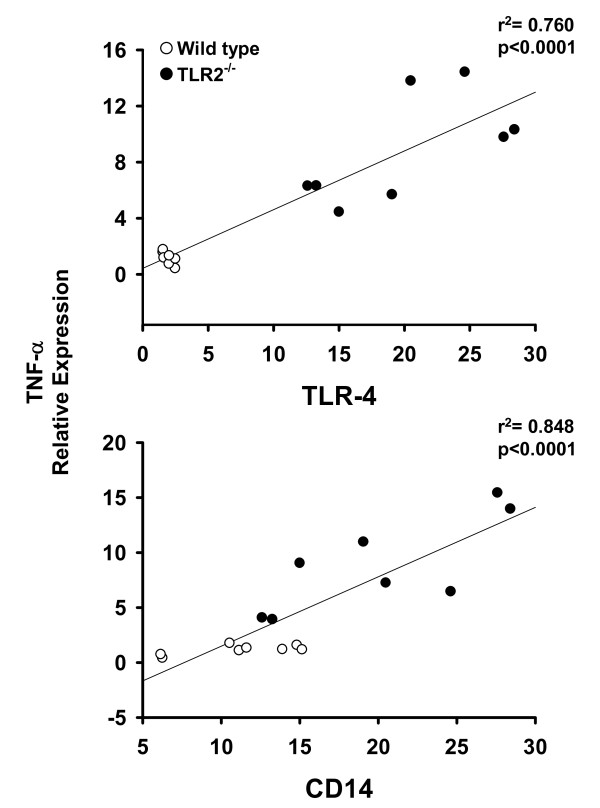
**Toll-like receptor-4 expression correlates with mRNA levels of TNF-α**. Real-time PCR data for TLR-4 and CD-14 were compared with the expression of the inflammation marker TNF-α using the Pearson's product moment statistical test. Each figure contains the compiled data from wild type and TLR-2^-/- ^mice fed PUFA. Correlation coefficients (p) and regression analysis (r^2^) are presented with each figure.

**Figure 10:**
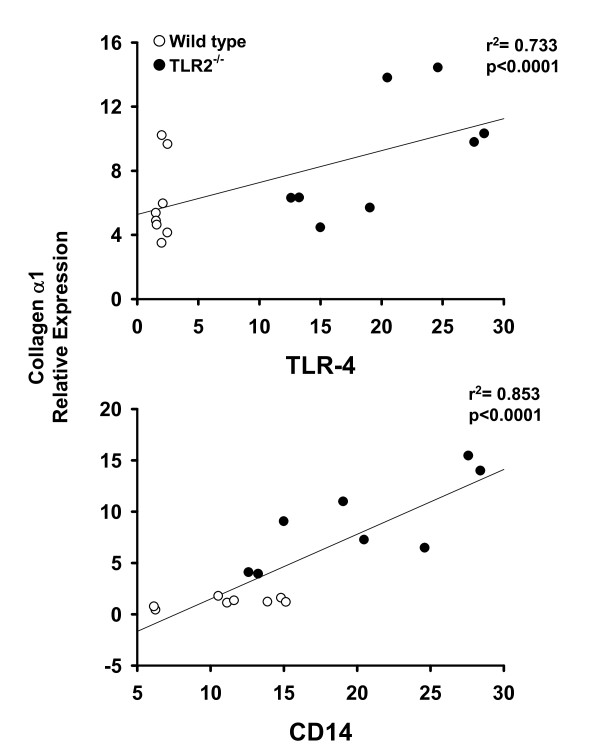
**Toll-like receptor-4 expression correlates with collagen α1 mRNA levels**. Real-time PCR data for TLR-4 and CD-14 were compared with the expression of the fibrosis marker of collagen α1 using the Pearson's product moment statistical test. Each figure contains the compiled data from wild type and TLR-2^-/- ^mice. Correlation coefficients (p) and regression analysis (r^2^) are presented with each figure.

The relationship between expression of TLR-4 signalling pathway components (i.e. TLR-4 and CD-14) and markers of inflammation and fibrosis was assessed using the Pearson's product moment statistical test. For this purpose, data from wild type and TLR-2^-/- ^mice fed PUFA were compiled. As shown in Figure 9, a significant positive correlation exists between the expression of TNF-α, and each of the TLR-4 signalling components. Feeding PUFA to both mouse strains also resulted in a significant positive correlation between TLR-4, CD14 and the expression of the matrix protein collagen α1 (Fig. 10). Similar results were obtained in wild type and TLR-2^-/- ^mice fed SAFA (data not shown).

## Discussion

A positive correlation exists between the incidence of metabolic syndrome and the amount of total fat consumed in the diet of obese humans. In the setting of obesity, endothelial dysfunction precedes the clinical manifestation of co-morbid diseases such as cardiovascular disease [21]. In contrast to the protective effects of unsaturated fat, feeding a diet rich in saturated fatty acids was shown to impair endothelial function as indicated by flow-mediated vasodilation, and stimulated inflammation as evidenced by increased P-selectin expression [22]. Although diet is known to contribute significantly to the genesis of cardiovascular disease, less is known about the influence of diet on the development of NASH. Thus, the goal of the first set of experiments described herein was to determine the effect of the quality of dietary fat on the extent of NASH. To this end, mice were fed a methionine- and choline-deficient diet that was enriched either in corn oil (PUFA) or coconut oil (SAFA). Because obese people in western cultures where liver disease is prevalent typically consume a diet that contains high amounts of saturated fat, we hypothesized that mice consuming SAFA would exhibit the greatest degree of NASH. Contrary to this hypothesis, histological and molecular evidence of NASH was significantly attenuated in this dietary group. These findings are similar to the reported influence of diet in rodent models of alcoholic steatohepatitis (ASH). For example, development of significant ASH in rodents requires the addition of polyunsaturated fatty acid to the ethanol-containing diet [23]. Nanji et al. (1989) were among the first to report that ethanol-containing diets high in saturated fat blunted the progression of ASH [11,24,25]. Moreover, saturated fat was shown to reverse fibrotic lesions that developed in late stages of ASH [25,26].

The protective effects of feeding a high saturated fat diet during ethanol exposure are believed to be mediated by adiponectin [27]. In fact, inclusion of purified saturated fatty acids in the culture medium of ethanol-treated 3T3 L1 adipocytes enhanced adiponectin promoter activity as well as expression, demonstrating a role for saturated fat in the production of this anti-inflammatory hormone [27]. Here, we show that feeding SAFA blunted increases in TLR-4 and CD14 expression in wild type mice, which is likely an additional protective benefit of high saturated dietary fat. The influences of fats in vivo are in stark contrast to the effects of fatty acids in vitro. For example, the major ω-3 unsaturated fatty acids found in fish oil eicosapentaenoic acid (EPA) and docosahexaenoic acid (DHA) blunted the inflammatory phenotype that results from the exposure of various human and murine cell lines to TLR-4 or TLR-2 agonists [8-11]. On the other hand, saturated fatty acids stimulated NFkB promoter activity and a pro-inflammatory phenotype [10]. It should be noted that these in vitro studies relied on the ectopic expression of TLRs; therefore, studies in cultured cells may not be representative of endogenous TLR responses in the setting of NASH.

In addition to diminished TLR-4 signalling components, feeding SAFA enhanced the expression of TLR-2 relative to the PUFA-fed mice. Therefore, the next experimental series examined the influence of TLR-2 deficiency on NASH pathogenesis. Despite partially overlapping ligand specificities and signalling pathways with TLR-4, data presented herein indicate that TLR-2 may play a protective role against the induction of steatohepatitis. Indeed, histological and molecular evidence of NASH were significantly enhanced in TLR-2^-/- ^mice relative to wild type mice, an effect most pronounced in mice fed SAFA. These current findings are consistent with a previous study by Szabo et al. that demonstrated enhanced sensitivity of TLR-2^-/- ^to NASH [28]. Compared to wild type mice, ALT was enhanced in TLR-2^-/- ^mice fed MCDD. Moreover, wild type mice with MCDD-induced NASH were more sensitive to the TLR-4 ligand lipopolysaccharide, but not TLR-2 ligands. Taken together with our present findings, these studies suggest that expression of TLR-2 plays a protective role against NASH pathogenesis. On the other hand, it must also be noted that the augmented injury in TLR-2^-/- ^mice reported herein was associated with enhanced expression of TLR-4 as well as the adaptor molecule CD-14. In fact, a significant positive correlation was found between elements of the TLR-4 pathway and the expression of pro-inflammatory and pro-fibrogenic mediators.

Injury observed using the MCDD dietary model of steatohepatitis is reportedly associated with increased intestinal permeability and the enhanced presence of endotoxin in the portal blood supply [14,29]. Previous findings using murine models of exposure to enteric bacterial pathogens and chemically-induced colitis indicated that TLR-2 plays a critical role in the maintenance of mucosal integrity. For example, Cario et al. demonstrated that stimulation of intestinal epithelial cells with a TLR-2 agonist preserved barrier function whereas mice deficient in TLR-2 expression exhibited disruptions in tight junctional complexes. Although not tested directly, these findings suggest that TLR-2 deficiency has the potential to augment steatohepatitis via promoting the escape of endogenous bacterial pathogens, which activate the TLR-4 signalling pathway.

In support of the idea that diets enriched in saturated fatty acid directly influence the fibrogenic response, Abergel et al. reported that exposure of stellate cells to palmitic acid significantly blunted transformation and the potential to produce matrix proteins such as type I collagen [30]. The response of stellate cells to saturated fatty acid was believed to be mediated via PPAR-γ. In fact, over-expression of this nuclear receptor prevented phenotypic transformation and production of collagen by stellate cells. We showed recently that feeding a high fat (coconut oil) diet to mice for 3 weeks enhanced PPAR-γ expression in the liver [31]. In the present study SAFA enhanced PPAR-γ expression in wild type mice, a phenomenon that was prevented in TLR-2^-/- ^mice. Moreover, an inverse relationship between the expression of collagen α1 and PPAR-γ in mice fed SAFA was observed. Thus, our finding of blunted PPAR-γ expression in livers of TLR-2^-/- ^mice suggests that this receptor plays a role in the induction of PPAR-γ in response to dietary saturated fat.

## Conclusions

Enrichment of the MCDD diet with saturated fat blunted NASH pathogenesis in wild type mice. The protective effects of saturated fat were less pronounced in mice deficient in TLR-2 expression, demonstrating the role of innate immunity in the response to the type of fat supplied in the diet. Clearly, more work is needed to understand mechanisms underlying this complex relationship between liver pathology and quality of dietary fat.

## Competing interests

The authors declare that they have no competing interests.

## Authors' contributions

CAR: Contributed the conceptual design, data acquisition, analysis and interpretation; was involved in manuscript preparation and has consented to the publication of this manuscript. LG: Contributed toward data acquisition, analysis and interpretation; was involved in manuscript preparation and has consented to the publication of this manuscript. MA: Contributed toward data acquisition, analysis and interpretation; was involved in manuscript preparation and has consented to the publication of this manuscript. JP: Contributed toward data acquisition, analysis and interpretation; was involved in manuscript preparation and has consented to the publication of this manuscript. KB: Contributed toward data acquisition, analysis and interpretation; was involved in manuscript preparation and has consented to the publication of this manuscript. PA: Contributed toward data acquisition, analysis and interpretation; was involved in manuscript preparation and has consented to the publication of this manuscript. KP: Contributed the conceptual design, data acquisition, analysis and interpretation; was involved in manuscript preparation and has consented to the publication of this manuscript.

## Pre-publication history

The pre-publication history for this paper can be accessed here:

http://www.biomedcentral.com/1471-230X/10/52/prepub
